# How Soil Scientists Help Combat Podoconiosis, A Neglected Tropical Disease

**DOI:** 10.3390/ijerph110505133

**Published:** 2014-05-13

**Authors:** Benjamin Jelle Visser

**Affiliations:** Academic Medical Center (AMC), University of Amsterdam, Meibergdreef 9, 1105 AZ Amsterdam, The Netherlands; Email: b.j.visser@amc.uva.nl; Tel.: +31(0)20-56-64400

Podoconiosis or “endemic non-filarial elephantiasis” is a tropical disease caused by prolonged exposure of bare feet to irritant alkaline clay soils of volcanic origin [1]. The name of the disease is derived from the Greek words for foot: *podos*, and dust: *konos*. Small mineral particles from irritant soils penetrate the skin and provoke an inflammatory response leading to fibrosis and blockage of lymphatic vessels, causing lymphoedema [2]. Patients suffer from disabling physical effects, but also stigma [1]. The disease can simply be prevented by avoiding contact with irritant soils (wearing shoes) but this is still an unaffordable “luxury” for many people. Podoconiosis is unique because it is a completely preventable non-communicable tropical disease [1]. In the past few years, podoconiosis has received increased advocacy and is now step by step appearing on the agenda of medical researchers as well as politicians. In February 2011, the World Health Organization (WHO) added podoconiosis to its lists of “neglected tropical diseases (NTDs)”. In March 2012, Prof. Gail Davey and colleagues launched “Footwork”, the International Podoconiosis initiative, which aims to facilitate public and private collaboration to control and eliminate podoconiosis. The associated website [3] provides a comprehensive overview of the epidemiology, economic, social and ethical consequences, diagnosis and clinical staging, aetiology and pathogenesis and treatment and care.

Historically, podoconiosis was widespread in Europe and North Africa too, but disappeared when the practice of wearing shoes became common. Podoconiosis nowadays affects an estimated 4 million people worldwide, and is most prevalent in the highland areas of Africa, but also found in mountainous areas in Central America and Asia [1,4,5]. A reliable and detailed map of the epidemiology of podoconiosis is needed to implement geographically targeted and cost-effective interventions in sub-Saharan Africa. Disease maps can help to identify critical and priority areas, and help to measure the association between environmental exposure factors and health events (for example, epidemiological podoconiosis studies). Identifying the ecological requisites for the distribution of the disease are key for the development of risk maps. A historical overview of the spatial distribution of podoconiosis in relation to environmental factors for Ethiopia was recently published [6], however, for most countries only scarce data, if any, exist.

**Figure 1:**
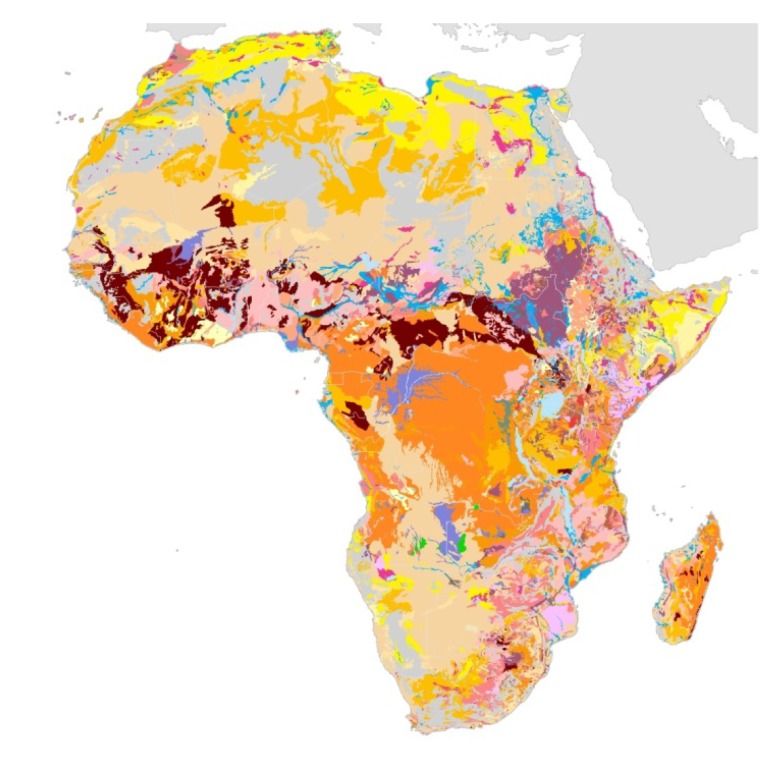
Map of Africa depicting different types of soil. Adapted from European Soil Portal—Soil Data and Information Systems [11].

Important ecologic factors with regard to podoconiosis are land surface temperature, mean annual precipitation, topography of the land and most importantly, the existence of irritant soils [5]. To facilitate research and decision making, relevant and up-to-date information on the state of soils is critical. Soil maps are already available for Europe, the Northern Circumpolar region, Latin America and Caribbean Islands and Asia [7]. This information was not available for most parts of Africa. A recent initiative of the European Commission’s Joint Research Centre, in collaboration with the African Union and the UN Food and Agriculture Organization, resulted in the first detailed and up-to-date soil Atlas of Africa [8]. Using state-of-the-art computer mapping techniques, the Soil Atlas of Africa (Figure 1) describes the different soil types that can be found in Africa. The African Soil Atlas can be a powerful tool to assist public-health professionals in better understanding the epidemiological complexities of podoconiosis and is highly relevant for the development of spatial mapping of podoconiosis by medical researchers. This English atlas is freely available and a French version is expected in 2014. It provides a very comprehensive interpretation of the significance of African soils. It must be noted however, that although the maps provide broad classifications of African soils, they do not present detailed descriptions of the chemical composition of the soil particles. Nevertheless the data in the African soil atlas provide a good basis for accurate and detailed soil maps for disease mapping and further geochemical research which needs to be focused on identifying the pathogenic particles in the volcanic soils that causes podoconiosis. Integrated disease mapping of other neglected tropical diseases that may overlap with podoconiosis (e.g., lymphatic filariasis), can result in more efficient utilization of resources and integrated disease control [9]. The emerging interdisciplinary field in which researchers study the relationship between natural geographic factors and their effects on human health is known as “medical geology” [10].

Also for other tropical disease [12], such as leishmaniasis [13], soil-transmitted helminths [14] (the roundworm (*Ascaris lumbricoides)*, the whipworm (*Trichuris trichiura*) and the hookworms (*Necator americanus* and *Ancylostoma duodenale*), bacteria (e.g., melioidosis) and fungi, soils are an important epidemiological factor. A useful map is the Global Atlas of Helminth Infections, which shows the geographical distribution of soil-transmitted helminthiasis, schistosomiasis (bilharzia), and lymphatic filariasis [15]. All the resources mentioned in this letter are available on an open access basis.
